# C5a receptor (CD88) promotes motility and invasiveness of gastric cancer by activating RhoA

**DOI:** 10.18632/oncotarget.12656

**Published:** 2016-10-14

**Authors:** Takayoshi Kaida, Hidetoshi Nitta, Yuki Kitano, Kensuke Yamamura, Kota Arima, Daisuke Izumi, Takaaki Higashi, Junji Kurashige, Katsunori Imai, Hiromitsu Hayashi, Masaaki Iwatsuki, Takatsugu Ishimoto, Daisuke Hashimoto, Yoichi Yamashita, Akira Chikamoto, Takahisa Imanura, Takatoshi Ishiko, Toru Beppu, Hideo Baba

**Affiliations:** ^1^ Department of Gastroenterological Surgery, Graduate School of Life Sciences, Kumamoto University, Kumamoto, Japan; ^2^ Department of Molecular Pathology, Graduate School of Life Sciences, Kumamoto University, Kumamoto, Japan

**Keywords:** C5a receptor, CD88, RhoA, gastric cancer, **Abbreviations:**C5aR, C5a receptor, GC, Gastric cancer, rC5a, recombinant human complement component C5a, GDP, guanosine diphosphate, GTP, guanosine triphosphate

## Abstract

**Purpose:**

Anaphylatoxin C5a is a strong chemoattractant of the complement system that binds the C5a receptor (C5aR). The expression of C5aR is associated with poor prognosis in several cancers. However, the role of C5aR in gastric cancer (GC) is unknown. The aim of this study was to examine the role of C5aR on GC cell motility and invasion.

**Experimental Design:**

The mechanism of invasion via C5aR was assessed by analyzing cytoskeletal rearrangement and RhoA activity after C5a treatment. Moreover, we investigated the relationship between C5aR expression and the prognosis of GC patients.

**Results:**

Two human GC cell lines (MKN1 and MKN7) had high C5aR expression. An invasion assay revealed that C5a stimulation promoted the invasive ability of MKN1 and MKN7 cells and that this was suppressed by knockdown of C5aR using siRNA or a C5aR-antagonist. Moreover, overexpression of C5aR in GC cells enhanced the conversion of RhoA-guanosine diphosphate (RhoA-GDP) to RhoA-guanosine triphosphate (RhoA-GTP) after C5a stimulation and caused morphological changes, including increased expression of stress fibers and filopodia. Examination of tumor specimens from 100 patients with GC revealed that high C5aR expression (35 of 100 samples, 35.0%) was associated with increased invasion depth, vascular invasion and advanced stage. The 5-year overall survival of patients with high or low C5aR expression was 58.2% and 88.5%, respectively (p=0.008).

**Conclusions:**

This study is the first to demonstrate that C5aR promotes GC cell invasion by activating RhoA and is associated with a poor prognosis in GC patients. Therefore, this study provides a biomarker for GC patients who require an advanced therapeutic strategy.

## INTRODUCTION

Gastric cancer (GC) is the third leading cause of cancer related death worldwide [1]. Despite advancements in our diagnostic and surgical capabilities and the discovery of new anti-cancer drugs for GC [1, 2], invasion and metastasis of cancer cells are still significant risk-factors for GC [3]. GC is associated with deep invasion into tissue and with lymphatic, vascular and peritoneal metastasis. The 5-year survival rate is approximately 5–20% in patients with advanced or metastatic GC [3, 4]. Despite several oncogenes and oncoproteins, such as human epidermal growth factor receptor 2, ras homolog gene family member A (RhoA), CD44v and E-cadherin, being associated with the proliferative and invasive ability of GC cells [5–9], only trastuzumab, which targets human epidermal growth factor receptor 2, is currently used clinically to treat advanced GC. Moreover, as few as 20–30% of all patients treated with trastuzumab have a clinical benefit [4, 10]. Therefore, new molecular targets are required that regulate the growth, invasive and metastatic ability of GC cells.

Anaphylatoxin C5a is a strong chemoattractant and an important factor of the complement system [11, 12]. The C5a receptor (C5aR) is a G-protein coupled receptor expressed by leukocytes [13]. C5a promotes leukocyte migration and their production of radical oxygen species by binding to the C5aR expressed on their cellular membranes, resulting in the initiation of inflammation [13, 14]. We previously reported that several solid cancer cells also express C5aR on their cellular membrane and that the C5a-C5aR axis promotes the invasiveness of cholangiocarcinoma by inducing actin reorganization and production of matrix metalloproteases [15]. It was also reported that C5aR expression in non-small lung cancer, breast cancer and ovarian cancer was associated with a poor prognosis [16–18]. However, the role of C5aR in GC is still mostly unknown. Here, we investigated the role of C5aR on the motility and invasive ability of GC cells *in vitro*. Moreover, we analyzed the relationship between C5aR-expression and the prognosis of GC patients.

## RESULTS

### Expression of C5aR in gastric cancer cell lines

**Figure 1:**
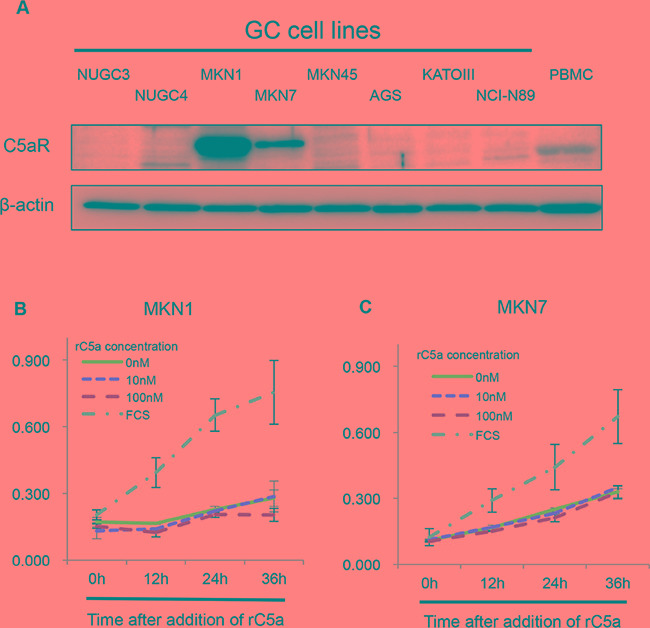
C5aR-expression in gastric cancer cell lines **A.**, Western blots demonstrating the level of C5aR-expression in gastric cancer cell lines. **B** and **C.**, growth assays using Cell Counting Kit-8 showing the growth of C5aR positive and negative gastric cancer cells following stimulating with rC5a. C5aR: C5a receptor, PBMC: peripheral blood mononuclear cell, rC5a: recombinant C5a, GC: gastric cancer.

We firstly investigated the expression of C5aR and C5a-like receptor 2 (C5L2, a second C5aR) in eight human GC cell lines. MKN1 and MKN7 cells had high-expression of C5aR and C5L2 (Figure 1A, Supplementary Figure S1). Next, we examined the growth ability of MKN1 and MKN7 cells when recombinant human complement component C5a (rC5a) was used to stimulate the cells C5aRs. The growth of MKN1 and MKN7 cells was not increased by rC5aR (Figure 1B and 1C).

### C5aR stimulation enhanced the invasiveness of cells with high-expression of C5aR

**Figure 2:**
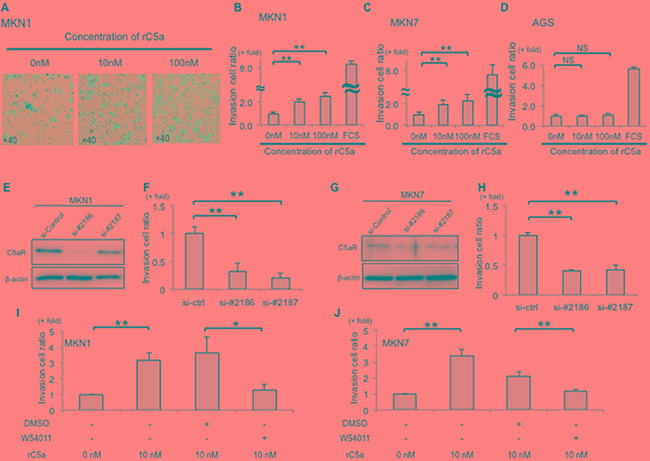
C5aR-stimulation with rC5a promotes invasion of gastric cancer cells **A.**, lower surfaces of an invasion membrane when MKN1 cells were assayed for invasion. **B** and **C.**, rC5a significantly enhanced the invasive ability of MKN1 and MKN7 cells. **D.**, rC5a did not enhance the invasive ability of AGS cells. **E–H.**, suppression of C5aR-expression using two kinds of siRNA significantly decreased the invasive ability of MKN1 and MKN7 cells. **I** and **J.**, W-54011, a C5aR-antagonist, significantly suppressed the invasive ability of MKN1 and MKN7 cells. C5aR, C5a receptor; rC5a, recombinant C5a; DMSO, Dimethyl sulfoxide; W-54011, C5aR-antagonist; *p<0.05; **p<0.01. NS, not significant.

To analyze the invasive ability of cancer cells, with or without high C5aR expression, cells were treated with rC5a and a Matrigel assay was performed. The invasiveness of MKN1 cells was enhanced 2.02-fold and 2.52-fold after treatment with 10 nM and 100 nM of rC5a, respectively, and the invasiveness of MKN7 cells was enhanced 1.93-fold and 2.28-fold, respectively (Figure 2A–2C). However, rC5a stimulation failed to increase the invasive ability of AGS cells that do not express C5aR (Figure 2D). Knockdown of C5aR-expression with C5aR siRNAs decreased the invasive ability of MKN1 and MKN7 cells (Figure 2E–2H). The C5aR antagonist W-54011 also suppressed the invasive ability of MKN1 and MKN7 cells by approximately 0.2–0.5 fold (Figure 2I and 2J). There was not significant difference in the growth ability of MKN1 and MKN7 cells with the knockdown of C5aR-expression using two different siRNAs of C5aR (Supplementary Figure S2A).

### C5aR stimulation enhanced the invasive ability and motility of C5aR overexpressing NUGC3 cells

**Figure 3:**
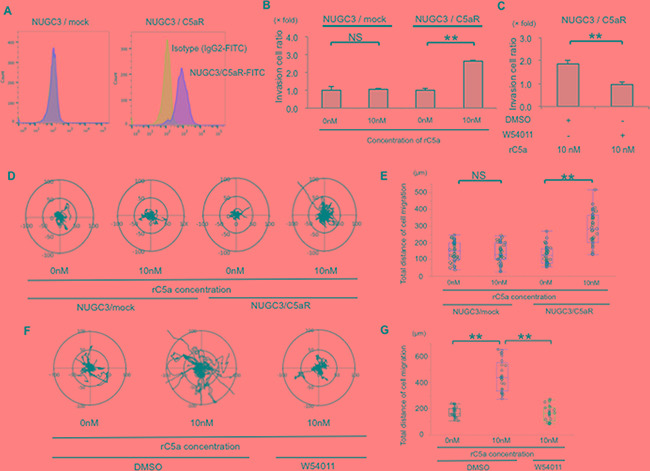
C5aR stimulation enhanced the invasive ability and motility of NUGC3 cell with C5aR-overexpression **A.**, Flow cytometry showed that C5aR proteins were overexpressed in the cellular membrane of NUGC3/C5aR cells. **B.**, rC5a significantly promotes the invasive ability of NUGC3/C5aR cells, but did not significantly promote the invasive ability of NUGC3/mock cells. **C.**, W-54011 significantly decreased the invasive ability of NUGC3/C5aR cells. **D** and **E.**, rC5a significantly promotes the mobility and the total distance of cell migration of NUGC3/C5aR cells but did not significantly promote the mobility of NUGC3/mock cells. **F** and **G.**, W-54011 significantly decreased the mobility of NUGC3/C5aR cells. C5aR, C5a receptor; rC5a, recombinant C5a; DMSO, Dimethyl sulfoxide; W-54011, C5aR-antagonist; **p<0.01. NS, not significant.

To further analyze the mechanism of the C5a to C5aR signal in human cancer cells, we overexpressed C5aR in C5aR-negative NUGC3 cells (Figure 1A). Flow cytometry using a C5aR-antibody confirmed C5aR expression on NUGC3/C5aR cells but not on NUGC3/mock cells (Figure 3A). The number of invasive NUGC3/C5aR cells was enhanced 2.62-fold after stimulation with 10 nM of rC5a (Figure 3B) and this was suppressed by W-54011 (Figure 3C). In addition, using real-time imaging, we evaluated the motility of NUGC3/C5aR cells after stimulating them with rC5a. Real-time imaging revealed that 10 nM of rC5a enhanced the total distance that the NUGC3/C5aR cells moved in Matrigel (Figure 3D and 3E) and that W-54011 suppressed the mobility of NUGC3/C5aR cells (Figure 3F and 3G).These results indicate that the C5a-C5aR signal enhances the invasion and mobility of GC cells *in vitro*.

### C5a-C5aR signal enhances RhoA-GTP production and changes the morphology of C5aR-expressing NUGC3 cells

**Figure 4:**
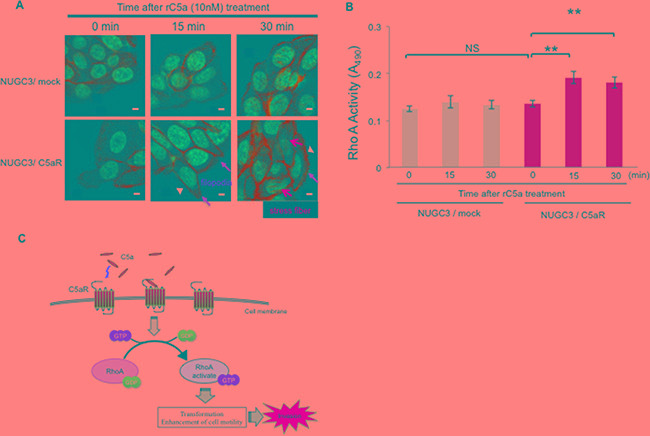
C5a-C5aR signaling enhances the production of RhoA-GTP and changes the cellular morphology of C5aR-expressing NUGC3 cells **A.**, NUGC3/C5aR and NUGC3/mock cells were incubated with rC5a (10 nM) and fixed at the indicated times. F-actin was visualized by immunofluorescence staining with Alexa 488–conjugated phalloidin. Scale bars, 10 μm. Orange and yellow arrows and arrowheads indicate filopodia, stress fibers and membrane ruffling, respectively. **B.**, analysis of the activation of RhoA using a G-LISA on the lysates of NUGC3/C5aR and NUGC3/mock cells were extracted at the indicated times after rC5a treatment. **C.**, diagram of C5a-C5aR signaling via the RhoA pathway in gastric cancer cells. C5aR, C5a receptor; rC5a, recombinant C5a; GDP, guanosine diphosphate; GTP, guanosine triphosphate; *p<0.05; **p<0.01. NS, not significant.

The morphologic change of cancer cells after rC5a stimulation was analyzed by immunofluorescence staining for F-actin. The majority of NUGC3/C5aR cells clearly showed strong filopodia after 15 minutes and strong stress fibers after 30 minutes of rC5a treatment (Figure 4A). Because activated RhoA stimulates the appearance of stress fibers and focal adhesions in quiescent cells and induces a change in cellular shape, [19–22] we hypothesized that the enhanced invasive ability following C5a stimulation was associated with RhoA activity. To test this, a Rho GTPase activity assay was performed. A significant conversion from RhoA-GDP to RhoA-GTP in NUGC3/C5aR cells was detected 15 and 30 minutes after rC5a treatment (Figure 4B). However, NUGC3/mock cells did not show any change in actin cytoskeleton or conversion of RhoA-GDP to RhoA-GTP after stimulation with rC5a. These results suggest that C5a-C5aR signaling enhances the conversion of RhoA-GDP to RhoA-GTP and causes morphological changes of gastric cancer cells.

### The relationship between the expression of C5aR and the prognosis of patients with gastric cancer

**Figure 5:**
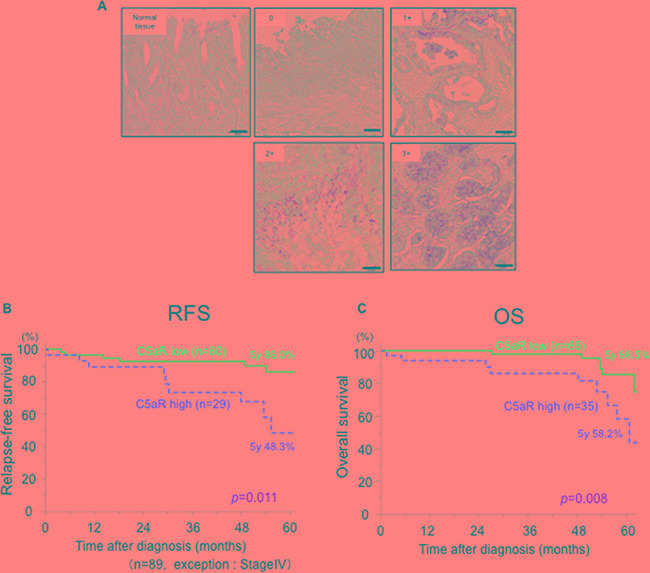
Relationship between C5aR expression and the prognosis of patients with gastric cancer **A.**, gastric cancer tissues were immunohistochemically stained with anti-C5aR antibody. One-hundred cases were scored from 0 to 3 according to the extent of C5aR staining in the cancer area. Scale bar: 100μm. **B.**, Relapse-free survival curves for 86 patients who underwent a gastrectomy for gastric cancer (excluding stage IV patients), which were stratified by low- and high-expression of C5aR. **C.**, Overall survival curves for 100 patients who underwent gastrectomy for gastric cancer, which were stratified by low- and high-expression of C5aR. C5aR, C5a receptor; RFS, relapse-free survival; OS, overall survival.

The relationship between the expression of C5aR and the prognosis of patients with GC was evaluated. C5aR was predominantly expressed on the cell membrane and a score from zero to three was assigned according to the extent of staining (Figure 5A). Scores of 0, 1, 2 and 3 were given to 54 (54.0%), 11 (11.0%), 16 (16.0%) and 19 (19.0%) of the patient samples, respectively. High C5aR expression (score of ≥2) was observed in 35 patients (35.0%), which was significantly associated with invasion depth, stage and vascular invasion (Table 1).

**Table 1:** Analysis of clinical factors associated with C5aR-expression from 100 patients with gastric cancer

Clinicopathological factors	C5aR-expression		*P*-value
	Low group (n=65)	High group (n=35)	
Mean age (years) ±SD	65.4±13.6	65.8±11.0	0.96
Sex			
Male / Female	40 / 25	24 / 11	0.48
Tumor location			
Upper / Middle / Lower	22 / 19 / 24	9 / 12 / 14	0.69
Differentiation			
Intestinal / Diffuse	30 / 35	15 / 20	0.75
Depth of invasion			
pT1-2	50	18	0.009
pT3-4	15	17	
Lymph node metastasis			
Negative	47	20	0.12
Positive	18	15	
pStage			
I / II / III / IV	47 / 8 / 5 / 5	14 / 10 / 5 / 6	0.02
Lymphatic invasion			
Positive / Negative / UK	40 / 24 / 1	20 / 15 / 0	0.60
Vascular invasion			
Positive / Negative / UK	37 / 27 / 1	12 / 23 / 0	0.02

UK: unknown

Sex, Tumor location, Differentiation, Depth of invasion, pN station, pStage, Lymphatic invasion, vascular invasion: χ^2^test, Mean age: unpaid test

The median follow-up period was 50.0 months. Among the 89 patients with stage I-III GC, the 5-year relapse-free survival rate for the patients with high C5aR-expression (n=29, 48.3%) was significantly worse than for those with low C5aR-expression (n=60, 86.0%; p = 0.011) (Figure 5B). From all 100 patients, the 5-year overall survival of the patients with high C5aR-expression (n=35, 58.2%) was significantly worse than those with low C5aR-expression (n=65, 88.5%; p = 0.008) (Figure 5C). A univariate analysis for prognostic factors for overall survival in 100 patients with GC identified sex, age, tumor depth, lymph node metastasis, vascular and lymphatic infiltration, differentiation and C5aR-expression (p ≤ 0.20). Multivariate analysis identified lymph node metastasis and C5aR-expression as prognostic factors for overall survival of the GC patients (Table 2).

**Table 2:** Univariate and multivariate analysis of prognostic factors associated with the overall survival of patients with gastric cancer

Factors	Univariate analysis		Multivariate analysis		
	5yOS (%)	*P*	HR	95% CI	*P*
Sex (Male / Female)	69.0 / 84.4	0.09			
Age (<65 / ≥65)	79.6 / 74.6	0.63			
pT1-2 / pT3-4	87.9 / 52.8	0.0002			
Lymph node metastasis −/+	87.7 / 53.7	0.0003	4.81	1.64-17.44	0.004
Vascular invasion −/+	86.0 / 60.5	0.009			
Lymphatic invasion −/+	95.0 / 59.9	0.003			
Differentiation I/D	84.1 / 69.4	0.11			
C5aR (Low / High)	88.5 / 58.2	0.004	3.13	1.12-9.44	0.02

OS: overall survival, HR: hazard ration, CI: confidence interval, I: intestinal type, D: diffuse type

## DISCUSSION

Complement C5a is a byproduct of the complement system via the classical, alternative and lectin pathways [11]. Recent studies indicate that C5aR (CD88) has a function in many different tissues, including the lung, heart, liver and kidneys [23–27], and that C5a is associated with many clinical conditions, including sepsis and rheumatoid arthritis [28–30]. We previously reported that C5a promoted the mobility and invasive ability of cancer cells, such as cholangiocarcinoma cells [15, 25]. Several papers have also reported relationships between C5aR expression and various cancers [16–18, 25]. Thus, the research related to C5aR in cancer and inflammation has recently received significant attention. In this study, we demonstrated that C5aR expressing GC cells have increased motility and invasiveness after stimulation with C5a. The C5a-C5aR signal enhanced the conversion of RhoA-GDP to RhoA-GTP and induced morphological changes to the GC cells. Moreover, examination of tumor specimens from GC patients who underwent a gastrectomy revealed that high C5aR-expression by the tumor is associated with a poor prognosis and increased invasiveness (Table 1, Figure 5). In addition, we reported that the gastric cancer patients with high-C5aR expression had higher incidence of liver metastasis than the patients with low-C5aR expression using another cohort (13.3% vs 3.9%, P=0.04) [31]. These results suggested that C5aR expressed on the membrane of GC cells may not only be a biomarker for prognosis but also an oncoprotein that promotes invasiveness by activating RhoA, resulting in liver metastasis and a poor prognosis (Figure 4C).

RhoA exists mostly in the cytosol in the GDP-bound form (Rho-GDP). When the appropriate extra cellular factors stimulate cells by binding to G-protein coupled receptors coupled to the heterotrimeric G12/13 protein, Rho-GDP is converted to Rho-GTP by the catalytic effect of guanine nucleotide exchange factors, several of which are direct targets of the G12/13 alpha subunits [19–22, 32]. This study is the first to demonstrate that aberrantly expressed C5aR promotes the conversion of RhoA-GDP to RhoA-GTP in cancer cells, which induces their cytoskeletal rearrangement and increases their invasive ability. *RHOA* encodes the small GTPase RhoA and regulates cancer cell contractility, cellular motility and metastasis of GC and other malignant tumors [5, 33–35]. Therefore, our clinical finding that high C5aR expressing tumors are more invasive, which includes deeper invasion depth and a higher rate of vascular invasion, compared with those with low C5aR expression is consistent with the results of these previous studies and with our *in vitro* study.

C5a production is necessary to activate RhoA via C5aR. It has been reported that C5a is generated by colon, ovarian and lung cancer cell without activation of the complement cascade [16, 17, 36]. In this study, GC cell lines also produced C5a at concentration as low as 60 pg/ml (Supplementary Figure S3). Moreover, it was reported that C5a was also released without activation of the complement cascade by cleavage of serum C5 by cancer cell membrane-bound proteases, thrombin or various proteases from phagocyte without activation of the complement cascade [27, 37, 38]. Therefore, C5a produced by both cancer cells and cleavage of serum C5 in the cancer microenvironment may continuously stimulate the conversion of RhoA-GDP to RhoA-GTP by stimulating C5aR, resulting in increased cancer cell motility and invasion.

C5a was reported to bind to not only C5aR, but also C5L2. In this study, we identified the expression of C5L2 in MKN1 and MKN7 cells (Supplementary Figure S1). It was difficult to evaluate the biological effect of C5L2 for gastric cancer cells, because siRNA used for C5aR knockdown also downregulated the C5L2 expression (Supplementary Figure S2B), It was suggested that C5L2 does not associated with cancer cell invasiveness via RhoA because C5L2 does not couple to G protein [12, 39, 40]. However, the effect of C5L2 on cancer cell biology cannot be ignored because stimulation of C5L2 with C5a or C5a-desArg activates extracellular signal-regulated kinase signaling via beta-arrestin [40]. Therefore, further study is needed to evaluate the association of C5L2 with cancer invasiveness.

*In vivo*, C5a is rapidly changed into C5a-desArg by carboxypeptidase enzymes. C5a-desArg has a binding affinity with C5aR that is 10- to 100-fold lower than that of C5a [12]. Little has been reported about the association between C5a-desArg and cancer cells. However, C5a-desArg induces Mac-1 induction in neutrophils via C5L2, resulting in an inflammatory reaction [41]. Because it was reported that Mac-1 promotes cancer liver metastasis *in vivo* [42], C5a-desArg may indirectly affect to cancer cell metastasis.

C5aR antagonist is an orally available C5aR inhibitor, which has been shown to competitively inhibit C5a binding and C5a-induced functions in human neutrophils [43]. The C5aR antagonist has been used as a novel therapeutic drug in the clinical setting for various inflammatory diseases, such as rheumatoid arthritis [44–46]. Here, we revealed that inhibition of the C5aR in GC cell lines using a C5aR antagonist suppressed the cell migration and invasion *in vitro* (Figure 3C–3G). Although a further *in vivo* study is required to clarify the utility of the antagonist, C5aR may be a potential target for the treatment of patients with GC.

The tumor microenvironment plays an important role in cancer cell invasion and metastasis. C5a-C5aR signaling in premetastatic organs promoted the generation of regulatory T cells and suppressed T-cell responses, which was followed by an increase in distal metastasis [47]. Piao C et al. showed that C5a promoted the infiltration of macrophages, neutrophils and dendritic cells by stimulating C5aR, and increased the expression of anti-inflammatory arginase 1, transforming growth factor β and interleukin 10 in the tumor microenvironment, promoting cancer metastasis [36]. Moreover, C5a generated in the tumor microenvironment promotes the production of vascular endothelial growth factor by endothelial cells, which promotes tumor angiogenesis [48, 49]. Thus, a C5aR antagonist might also suppress the invasion or metastasis of cancer cells by inhibiting C5a-C5aR signaling in cells of the tumor microenvironment.

In conclusion, we demonstrated that C5aR stimulation with C5a promotes the motility and invasion ability of GC cells by activating RhoA. Moreover, patients with high C5aR-expression were associated with a more invasive tumor and a poor prognosis. This study provides a novel insight into the mechanisms of GC cell invasion through the complement system and identifies a potential GC patient population that requires an advanced therapeutic strategy.

## MATERIALS AND METHODS

### Cell lines

We obtained the human GC cell lines MKN1 (established from adenosquamous carcinoma), MKN7 (established from well-differentiated tubular adenocarcinoma), NUGC3 and AGS (established from poorly differentiated tubular adenocarcinoma) from ATCC (Manassas, VA). Each cell line was grown in RPMI-1640 medium (Sigma-Aldrich, St. Louis, MO) supplemented with 10% decomplemented fetal bovine serum. The cells were maintained at 37°C and 5% CO_2_.

### Reagents

A rabbit polyclonal antibody against C5aR (CD88; sc-25774) was purchased from Santa Cruz Biotechnology (Texas, USA). An antibody against C5L2 was purchased from Atlas Antibodies (Stockholm, Sweden). A FITC-conjugated mouse monoclonal antibody against C5aR (MCA2059FT) and a FITC-conjugated mouse polyclonal antibody against IgG2a (STAR133F) was purchased from Bio-Rad (California, USA). A mouse monoclonal antibody against C5aR (HM2094) was purchased from Hycult Biotech (PB Uden, Netherlands). Recombinant human Complement Component C5a (rC5a; 2037-C5-025) was obtained from R&D Systems (Minneapolis, USA). The C5a receptor antagonist, W-54011 (sc-203863) was purchased from Santa Cruz Biotechnology. An ELISA kit was purchased from Thermo Scientific (Frederick, USA).

### siRNA transfection

C5aR-expression was transiently reduced using a predesigned Silencer Select siRNA directed against C5aR (Life Technologies Japan Ltd, Tokyo, Japan), and a non-targeting siRNA was used as a negative control. We transfected GC cells with the annealed siRNA for 48 hours using Lipofectamine RNAiMax (Thermo Fisher Scientific, Yokohama, Japan). The downregulation was confirmed by western blot analysis using a C5aR antibody.

### Western blot analysis

Cultured cells were washed in PBS, lysed using RIPA Lysis and Extraction Buffer (Thermo Fisher Scientific, Yokohama, Japan) and added to a protease/phosphatase inhibitor cocktail (Thermo Fisher Scientific, Yokohama, Japan). We subjected protein samples to sodium dodecyl sulfate polyacrylamide gel electrophoresis and transferred it to nitrocellulose membranes. The membranes were blocked with 5% low-fat dry milk in Tris-Buffered saline and Tween (25 mM Tris [pH 7.4], 125 mM NaCl and 0.4% Tween) and then incubated with the primary antibodies, including anti-C5aR and anti-β-actin antibodies, which were diluted in Tris-Buffered saline and Tween. The samples were incubated at 4 °C overnight. Signals were detected by incubation with rabbit secondary antibodies at room temperature for an hour using the ECL Detection System (GE Healthcare, Little Chalfont, UK). Peripheral blood mononuclear cells were used as positive control for C5aR and C5L2.

### Establishment of C5aR stably expressing NUGC3 cells

Human C5aR cDNA was purified according to a previously reported method [15]. Briefly, full-length human C5aR cDNA (1,053 base pairs) was amplified by PCR using a human macrophage cDNA library and the cDNA was subsequently subcloned into pENTR/D-TOPO vectors (Thermo Fisher Scientific, Yokohama, Japan). After confirming the sequence, the cDNA was inserted into pCAG-IRES-puro vectors using the Gateway system (Invitrogen). The purified plasmid of C5aR was transfected into NUGC3 cells using Lipofectamine 3000 (Thermo Fisher Scientific, Yokohama, Japan). After transfection, the cells were cultured with selection medium supplemented with puromycin (1 mg/ml) for 2 weeks (NUGC3/C5aR). NUGC3 cells transfected with empty-pCAG-IRES-puro vectors were used as a control (NUGC3/mock).

### Growth assay

MKN1 and MKN7 cells were inoculated in a 96-well plate at 2.0×10^3^ cells in 100 μl per well and the plate was incubated overnight in a humidified incubator at 37°C with 5% CO_2_. The medium was exchanged with medium containing the indicated concentrations of rC5a. Each well of the plate also received 10 μl of the Cell Counting Kit-8 solution (Dojindo Molecular Technologies, Kumamoto, Japan) at the indicated time. The absorbance was measured at 450 nm using a microplate reader after incubating the plate for 1.5 hours. The absorbance of each sample was measured in triplicate.

### Invasion assay

BioCoat Matrigel invasion chambers were used (24-well plates, 8 μm pores; BD Biosciences, California, USA) according to the manufacture's protocol. MKN1, MKN7 and NUGC3/C5aR cells (5.0×10^4^) were suspended in RPMI-1640 and seeded into the upper chamber. RPMI-1640 supplemented with rC5a or a carrier solution (PBS) was placed in the lower chamber. The C5aR-antagonist (W-54011) or a carrier control (dimethyl sulfoxide) was added to the cell medium at the indicated concentrations 3 hours before the cells were seeded into the upper chamber. A C5aR-antagonist (W-54011) or carrier control (dimethyl sulfoxide) was also added to the medium of the upper chamber. The number of cells that migrated through the membrane were counted in five microscopic fields (×20 magnification) per membrane. The average was calculated from triplicate samples and statistical analyses were performed by two-tailed t-tests.

### Flow cytometry analysis

The cells were adjusted to a concentration of 1.0×10^7^ cells/ml in PBS with 2% fetal bovine serum. Cell suspensions were incubated with a mouse monoclonal anti-C5aR-FITC antibody (MCA2059FT) or a mouse polyclonal anti-IgG2a-FITC antibody (STAR133F) for 30 minutes at 4°C. Flow cytometry analysis was performed with a FACSVerse (BD Biosciences) and data were analyzed with FlowJo 3.3 software (Tree Star, San Carlos, CA).

### Real-time imaging of cell migration

We performed real-time imaging of GC cells in 6-well plates. Each well was coated with 200 μl of BD Matrigel (BD Biosciences, California, USA). NUGC3/C5aR cells were plated and allowed to adhere for 12 hours. The 6-well plates were imaged with a KEYENCE BZ-X700 all-in-one fluorescence microscope equipped with a CO_2_ and temperature controlled chamber and time-lapse tracking system (KEYENCE, Osaka, Japan). Phase contrast images were taken every 10 minutes for 24 hours and converted to movie files using a BZ-X Analyzer (KEYENCE). We analyzed the movies for cell migration with the video editing analysis software VW-H2MA (KEYENCE) and subsequently processed the tracking data with Microsoft Excel 2010 (Microsoft, Redmond, WA) to create xy coordinate plots and distance measurements.

### Immunofluorescence analysis

Cells (5.0×10^4^) were seeded on a 35 mm glass-bottomed dish (Matsunami Glass Ind., Kishiwada, Japan) and allowed to grow for 24 hours. After 6 hours of serum starvation, the cells were stimulated with 10 nM of rC5a for the stated time periods. The cells were then fixed in 4% paraformaldehyde for 15 minutes, permeabilized in 0.1% Triton X-100 for 5 minutes, incubated with 5 U/ml Alexa 488-phalloidin (Invitrogen Life Technologies) for 40 minutes and then washed with PBS. Cell nuclei were counterstained with Hoechst 33258 (Life Technologies, Tokyo, Japan) for 15 minutes. Images were obtained and processed with a FluoView 1200 laser scanning confocal microscope (Olympus, Tokyo, Japan).

### Rho GTPase activity assays

RhoA activity was evaluated using a colorimetric RhoA activity assay (Cytoskeleton, Inc., Denver, USA) according to the manufacturer's instructions. Briefly, cells were lysed using an ice cold lysis buffer and the protein concentration was equalized. We applied 25 μl of equalized cell extract on the kit provided 96-well plate and placed the plate on an orbital shaker at 400 rpm at 4°C for 30 minutes. The plate was subsequently incubated at room temperature in Antigen Presenting Buffer for 2 minutes, anti-RhoA primary antibody for 45 minutes, the secondary antibody for 45 minutes, and horseradish peroxidase detection reagent for 15 minutes. The absorbance of each sample was measured in triplicate at 490 nm and recorded using a microplate spectrophotometer.

### ELISAs of cultured medium supernatant

The concentration of C5a was evaluated using an enzyme-linked immunosorbent assay (Thermo Scientific, Frederick, USA) according to the manufacturer's instructions. Briefly, medium of the indicated cells were taken, when the cells were 80-90% confluent. Cell supernatant (100μL) was added to the wells and incubated for 2.5 hours at room temperature with gentle shaking. Each well received 100μL of biotinylated antibody and the place was incubated for 1 hour at room temperature with gentle shaking. Streptavidin-HRP solution (100μL) was added to each well and the place was incubated for 45 minutes at room temperature with gentle shaking. Each well then received 100μL of TMB Substrate and the place was incubated for 30 minutes at room temperature in the dark with gentle shaking. The absorbance of each sample was measured in triplicate at 450 nm and recorded using a microplate spectrophotometer.

### Patients

The surgical GC specimens were obtained from 100 patients who underwent gastrectomy in Kumamoto University Hospital (Kumamoto, Japan) from January 2010 to December 2011. A follow-up, including an upper gastrointestinal endoscopy, computerized tomography and blood examination, was conducted every 3 months for less than 2 years after surgery. We performed the follow-up every other year from a half year until 5 years after surgery. Diagnosis of GC, invasion depth, vascular and lymphatic invasion, lymph node metastasis and differentiation were confirmed by histopathological examination of the resected specimens. Written informed consent was obtained from each subject, approval was acquired from the institutional ethics committee (No. 1051) and the study was performed in accordance with the Helsinki Declaration of 1975. Tumor staging followed the American Joint Committee on Cancer Staging Manual (7th edition) [50].

### Immunohistochemistry

Sections 4 μm thick were pretreated with 0.3% H_2_O_2_ in methanol for 30 minutes after deparaffinization. Sections were then incubated with diluted primary antibody (C5aR) at 4 °C overnight and detection was performed with an EnVision+ detection system (Daka, Tokyo, Japan). Nuclei were counterstained with hematoxylin. All staining was independently scored by two blinded pathologists. For each tissue section, five high-power fields (×100) were randomly selected and the average stained area per each power field were calculated. The average percentage of the stained area was scored as 0 for 0%, 1 for 1–25%, 2 for 26–50% and 3 for 51–100%. A total C5aR expression score of ≥2 was classified as high C5a expression.

### Statistical analysis

All statistical analyses were carried out using JMP® 11 (SAS Institute Inc., Cary, NC, USA). Continuous variables were compared using a Student's t-test. Categorical variables were compared using the chi-square test. Survival time was defined from the date of diagnosis until the date of death; overall survival and relapse-free survival were calculated using the Kaplan–Meier method and differences in survival between the groups were analyzed using a log-rank test. Variables that exhibited *P* value of <0.20 on univariate analysis for risk factors affecting OS were subjected to multivariate analysis using Cox proportional hazard model**.** All variables associated with prognosis were candidates using a stepwise backward elimination procedure with a threshold *P* *<*0.05. p<0.05 was considered significant.

## SUPPLEMENTARY MATERIALS FIGURES AND TABLES


