# Supplementary material for: C5a receptor (CD88) promotes motility and invasiveness of gastric cancer by activating RhoA
Source: Oncotarget. 2016 Oct 14;7(51):84798–809. doi: 10.18632/oncotarget.12656 (PMC5356699; doi:10.18632/oncotarget.12656)
Supplement: Supplementary file 1 [file oncotarget-07-84798-s001.pdf]

C5a receptor (CD88) promotes motility and invasiveness of gastric cancer by activating RhoA

SUPPLEMENTARY FIGURES

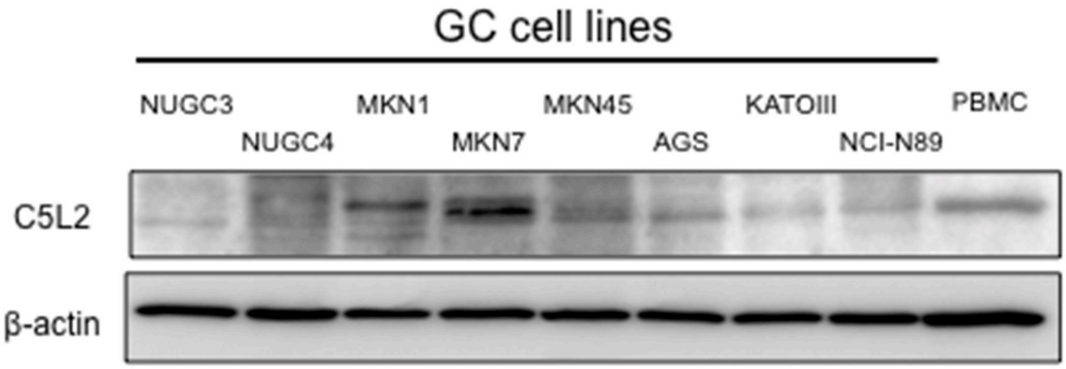

Supplementary Figure S1: C5L2-expression in gastric cancer cell lines. Western blots demonstrating the level of C5L2-expression in gastric cancer cell lines. C5L2: C5a like receptor 2, PBMC: peripheral blood mononuclear cell.

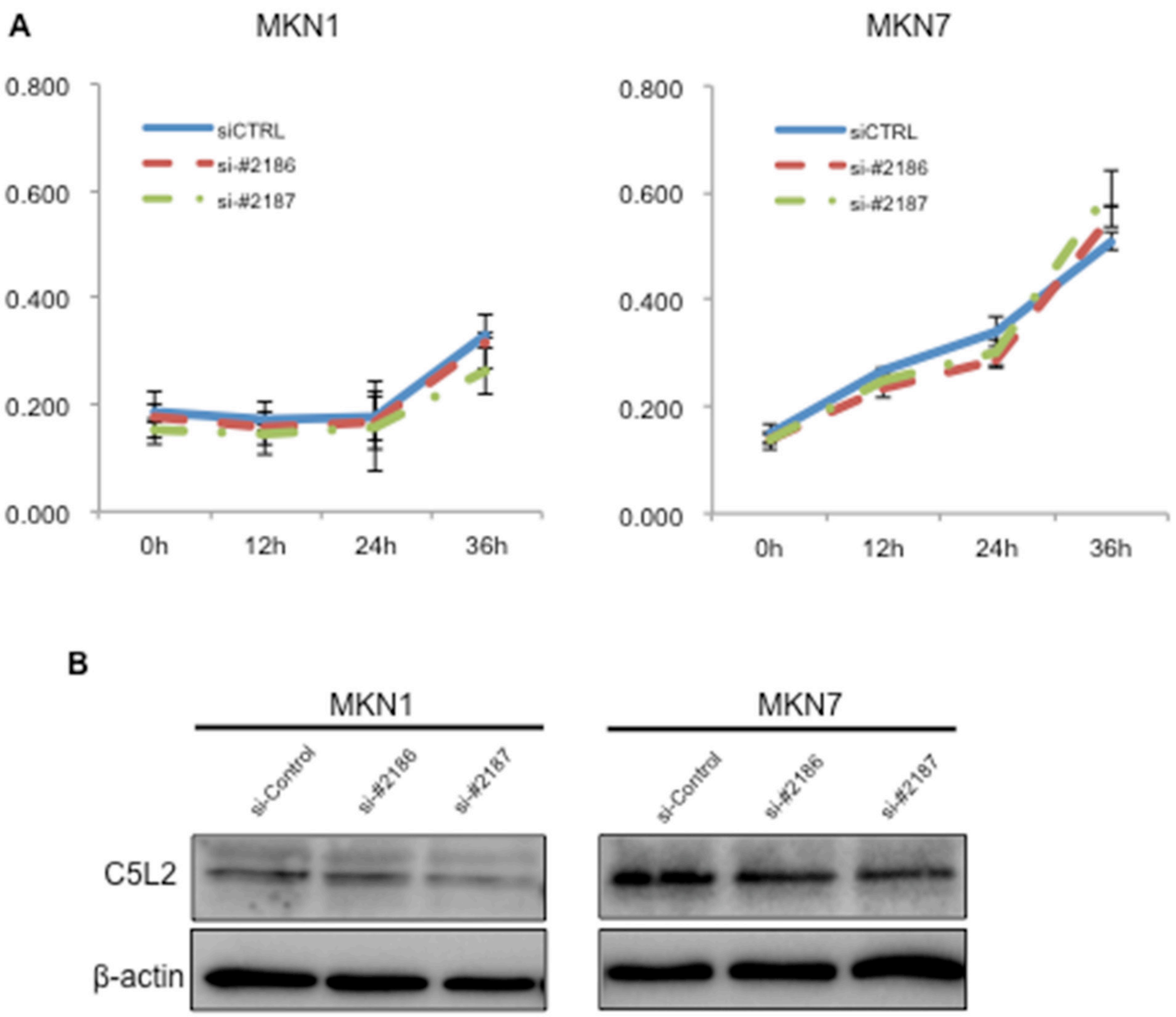

Supplemental Figure S2: Suppression of C5aR-expression and change of C5L2- expression using two kinds of siRNA in gastric cancer cell lines. A., Growth assays using Cell Counting Kit-8 showing the growth of MKN1 and MKN7 cells inhibited C5aR-expresion using two kinds of siRNA. **B.**, Change of C5L2-expression by siRNA used for C5aR knockdown (si-#2186, 2187). C5aR: C5a receptor, C5L2: C5a like receptor 2.

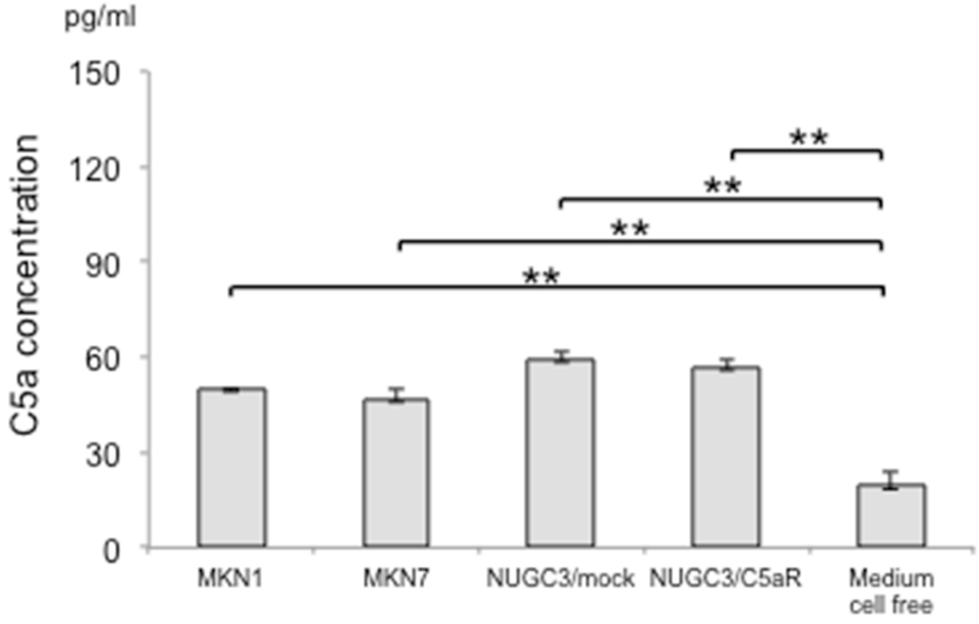

Supplemental Figure S3: Concentration of C5a in medium that cultured gastric cancer cells. Analysis of the concentration of C5a in cultured medium using an ELISA. ELISA: enzyme-linked immunosorbent assay.
